# Migrants face Barriers to Obtaining Electronic Identification: A population-based Study Among Older Russian-speakers in Finland

**DOI:** 10.1007/s10916-023-01940-5

**Published:** 2023-04-01

**Authors:** Laura Kemppainen, Sirpa Wrede, Anne Kouvonen

**Affiliations:** 1 Faculty of Social Sciences, University of Helsinki, P.O. Box 4, Yliopistonkatu 3, 00014 Finland; 2 Centre for Public Health, Institute of Clinical Science, Queen’s University Belfast, Royal Victoria Hospital, Block A, Belfast, BT12 6BA Northern Ireland

**Keywords:** Electronic identification, Health services, Older migrants, Digital inequalities, Russian migrants, Finland, Survey study

## Abstract

As digital technologies continue to transform health care and health systems, they will continue to have a lasting impact on health services. Many health and social care services have rapidly become ‘digital by default’. The electronic identification (e-ID) technology is needed for secure authentication to digital services. Recent studies have shown that the ‘digital divide’ is prominent between ethnic minorities and the majority populations and between older and younger adults. Inequalities related to not having an e-ID, which is in many countries required to access digital health services, remain under-researched. Moreover, there is a lack of knowledge of the use of digital services among older migrants. This study analyses general socio-demographic as well as migration specific factors that may be associated with not having an e-ID among older migrants. We used the Care, Health and Ageing of Russian-speaking Minority in Finland (CHARM) study, which is a nationally representative survey of community-dwelling Russian-speaking adults aged ≥ 50 years living in Finland (N = 1082, 57% men, mean age 63.2 years, standard deviation 8.4 years, response rate 36%). Our results showed that 21% of older Russian-speakers did not have an e-ID. Our regression analysis showed that older age and poorer economic situation were associated with a lower probability of having an e-ID. In addition, we found an association between not speaking local languages and not having an e-ID. This may relate to private banks regulating the requirements for obtaining the most common e-ID method, online banking ID. We argue that for individuals who are already in vulnerable positions, current e-ID practices might pose yet another obstacle to obtaining the health services they need and are entitled to.

## Background

Digital transformation has had a substantial impact on health care and health care systems and will continue to have a lasting effect on health services in the future [1]. Digital information and communication technologies (DIT) are becoming increasingly important for public administration of welfare states. Due to enthusiastic digitalisation projects, many welfare state services have rapidly become ‘digital by default’ [2–7]. The Covid-19 pandemic has catalysed the use of digital technologies in health and social care provision, which has exacerbated existing digital inequalities [8–12].

As a Nordic welfare state, Finland provides extensive social rights and a publicly provided health system financed by taxes [13]. Subsequent Finnish governments have been promoting digitalisation as an overarching goal in all policy sectors and implementing large-scale digitalisation projects in the public sector, including health services. The *My Kanta* portal, which can be accessed with an e-ID, constitutes a comprehensive digitalisation of personal health records and prescriptions. In addition, many municipalities and private sector health service providers have introduced digital appointment booking systems, service chats, and remote consultations, which most require an e-ID. Digitalisation of services demands a more active position from the service user than face-to-face services administered by professionals, and this may pose problems to already disadvantaged groups, which typically also need these services more [5, 14]. Moreover, people who are in vulnerable or marginalised position often have a poorer access to DIT, which can lead to reduced access to health services and health information [5, 9, 15, 16]. Older adults [17–19] and people from racial/ethnic minorities and migration backgrounds [20–23] have been shown to be at greater risk of digital exclusion. Weak local language skills, low educational level, and low socio-economic status, and living in segregated neighbourhoods have been shown to increase the risk of digital exclusion and explain the divide between migrant and non-migrant people [24–27]. Older adults from migration backgrounds may face a double jeopardy in regards to these digital access barriers [21, 22, 28].

One central aspect of digitalisation of public services is related to the protection of personal information when accessing digital services. Thus, the development of electronic identification (e-ID) technology is central in digitalisation projects, as it is a prerequisite for secure authentication to digital services [29–31]. In Nordic countries, the advanced digital identification infrastructure is regarded as one of the main contributors to their success in being the most advanced digital economies in Europe [7, 32]. In Nordic countries, including Finland, the digital identification systems that are used in digital public services have been created in close partnership with the private financial sector and they rely mainly on online banking identification methods [32–34]. While the state has relied on the market-procured identification methods to save on innovation and development costs, the banking sector has created a strong status quo as the owner of these e-ID methods [33, 34]. In Finland, the main method of strong electronic identification in the public services is online banking ID, which is used by 90% of the adult population. Other methods, that is, mobile certificate or a state issued e-ID card, are used by only 8% and 2% of the adult population, respectively [35]. For electronic identity authentication, online banking IDs are popular because they are easy to use and widely available. Nowadays, most banking activities are conducted online, so in practice an online banking ID is essential. In addition, online banking does not require any extra device, whereas the use of the state issued ID-cards requires a specific card-reader.

As banks have the right to choose their customers according to their own principles of privacy and security and the requirements of related legislation, the banking sector has acquired a gatekeeper role in regard to the most used e-ID platforms. In Finland, the bank’s requirements for obtaining online banking details (i.e., e-ID) includes having a Finnish personal ID number, a proof of regular earnings or social benefits, and sufficient local language (Finnish or Swedish) skills. These requirements may create problems of access to e-ID for people in disadvantaged positions, including many people with migration backgrounds. Consequently, the non-discrimination ombudsman in Finland has received complaints from migrants who have not been able to open bank accounts and acquire online banking ID [36].

As the online banking ID is used as the identity authentication requirement for digital public health services, not having an e-ID creates a significant barrier of access to these services. A recent study showed that 98% of the Finnish born working-age population have an e-ID, while 89% of people who are 55–74 years and 57% of those who are older than 74 years have it [37]. Among working-age migrants, the figure is 88% [38]. There were significant differences in access to e-ID between different migrant groups. Whilst 98% of migrants from the EU, EFTA and Northern European countries have e-ID, only 75% of those from the Middle East and Northern Africa have it [38]. However, there are no studies examining this phenomenon among older migrants.

This study is set to investigate access to an e-ID among Russian-speakers aged 50 or older in Finland. Russian-speaking migrants are the biggest foreign-born language group in Finland, comprising of over 84,000 speakers and over one fifth of all foreign language speakers [39]. The research questions are:

1. What are the socio-economic determinants of not having an e-ID?
2. Are there migrant specific factors of not having an e-ID, such as length of stay, citizenship status or language skills?

## Data and Methods

The data were drawn from the Care, Health and Ageing of Russian-Speaking Minority in Finland (CHARM) survey that was collected in 2019 [22, 40]. The target population of the survey was Russian-speaking community-dwelling adults, who are 50 years of age or older and who permanently reside in Finland. The study was designed to collect data on participants’ health and well-being, public service experiences, digital inclusion, and access to different types of care. A random sample of 3000 people was drawn from the register of the Digital and Population Data Services Agency; their register covers all persons registered as permanently living in Finland. The sample was stratified by gender. Response rate was 36% (N = 1082; 57% men and 43% women; mean age 63.2 years, standard deviation 8.4 years). The questionnaire was available in Russian and Finnish and there was an opportunity to answer online. However, only approximately 8% answered online. Survey weighting was used to account for the sex-based stratification of the sample. An auxiliary gross sample of information from national registers (sex, age, income, pensions, unemployment, and region) was used to correct non-response bias. Study participation was voluntary, and the participants were informed of their right to withdraw at any time without any consequences. The Ethical Review Board in the Humanities and Social and Behavioural Sciences at the University of Helsinki approved the study protocol (#6/2019).

The dependent variable was dichotomous, so we used logistic regression analysis. The results from the logistic regression models are presented as average marginal effects (AMEs), which can be interpreted as predicted probabilities and compared across regression models [41].

The outcome variable was based on the survey question on whether or not the participant has in their personal use online banking ID, or mobile identification for electronic identification (yes/no). Participant’s sex and age were acquired from the sample drawn from the Finnish population register and other variables are from the survey. Basic socio-economic variables included household’s monthly net income in four categories (less than 1000€ / 1000–1499€ / 1500–2499€ / over 2500€ (reference category)) and receiving of means-tested income support during the last year (recipient / non-recipient (ref.)). Education received in Finland was categorized as having post-secondary education in Finland (vocational school or higher education, yes / no (ref.)). Due to the larger proportion of higher education degrees in the country of origin, the variable on education from the country of origin was categorized as having higher education or not (yes / no (ref.). Participants were asked whether they had in their personal use a smartphone, tablet computer, or a laptop. The variable denotes whether they have one or more of these (yes (ref.) / no). Migration specific variables were the length of stay in years (continuous), having Finnish citizenship (yes (ref.) / no) and local language (Finnish or Swedish) skills in three categories (cannot speak at all, basic level, advanced level (ref.)). The logistic regression analysis was conducted with Stata 17 software. A p-value of < 0.05 was considered statistically significant.

## Results

Table 1 describes the characteristics of the sample and shows that over 55% reported that their household net income was less than 1500 Euros per month. Consequently, approximately 40% of the participants had received means tested income support during the last year. This is an indicator of widespread poverty in this group as in 2020 the corresponding figure for the whole population of Finland was only 8% [42]. While 50% of the participants had received higher education in their country of origin, almost 64% did not have any Finnish education. Participants had lived in Finland on average 17 years (range 0 to 76 years) and about half of them had a Finnish citizenship. Only less than 6% did not have smartphone, tablet computer or smartphone in their personal use.

**Table 1:** Characteristics of the sample (n = 1082)

	n	%	Total		
**E-ID**					
No	202	21.6	937		
Yes	735	78.4	937		
**Sex**					
Female	466	43.1	1082		
Male	616	56.9	1082		
**Age**					
50–64	653	60.4	1082		
65–90+	429	39.7	1082		
**Monthly household income**					
Less than 1000€	322	30.5	1056		
1000–1499€	260	24.6	1056		
1500–2499€	287	27.2	1056		
over 2500€	187	17.7	1056		
**Income support**					
Non-recipient	421	38.9	1082		
Recipient	592	54.7	1082		
Missing	69	6.4	1082		
**Education in Finland**					
None	688	63.6	1082		
Some	394	36.4	1082		
**Higher education in the country of origin**					
Yes	541	50.0	1082		
No	541	50.0	1082		
**Personal smartphone, tablet or laptop**					
No	60	5.7	1056		
Yes	996	94.3	1056		
**Finnish citizenship**					
No	546	51.0	1071		
Yes	525	49.0	1071		
**Local language skills (FIN/SWE)**					
Cannot speak	117	10.8	1082		
Basic level	517	47.8	1082		
Advanced level	385	35.6	1082		
Missing	63	5.8	1082		
	**Mean**	**Std.D**	**Min**	**Max**	**Total**
**Length of residence**	17.4	9.1	0	76	1056

**Fig. 1:**
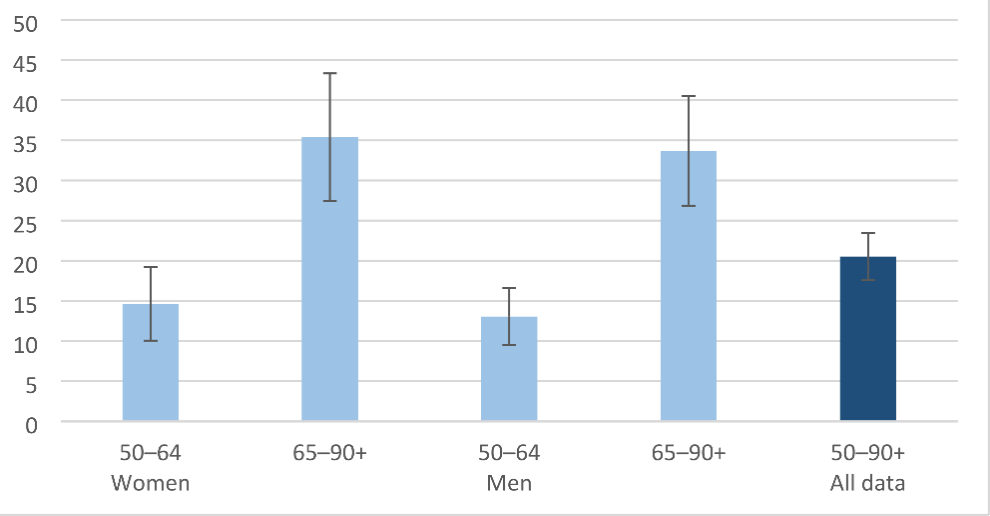
Percentages and 95% confidence intervals of not having an e-ID by age group

Figure 1 shows the weighted percentages with 95% confidence intervals of not having an e-ID by age group. In the total population of Russian-speaking migrants aged 50 years or older, nearly 21% (95% CI: 18–24%) did not have an e-ID. In those aged 65 years or older, 35% (95% CI: 27–43%) of women and 34% (95% CI: 27–41%) of men did not have an e-ID, while in those aged 50 to 64 years these figures were nearly 15% (95% CI: 10–19%) for women and 13% (95% CI: 10–17%) for men, respectively. We additionally counted the proportion of e-ID holders in 55–74-year-olds and over 74-year-olds to be able to compare the proportions to those of the general Finnish population in these age groups. In these groups, the proportion of participants who did not have an e-ID were 20% (95% CI: 17–24) and 48% (95% CI: 36–61%), respectively (data not shown).

In Table 2, we present the results of our logistic regression as average marginal effects. The bivariate model showed that older age, lower income, receiving income support, not having an access device (smartphone, tablet, or computer), not having Finnish citizenship and poorer command of local languages (Finnish or Swedish) were associated with a higher probability of not having an e-ID. The lack of Finnish education was also associated with not having an e-ID. Participant’s sex, education in the country of origin and the length of residence were not statistically significantly associated with the outcome in the bivariate model.

In the full model, sex, higher education in the country of origin, and length of residence remained non-significant. Having Finnish education and Finnish citizenship lost statistical significance after adding the other variables. The full model shows that people aged 65 or older were more likely to not have an e-ID than the younger cohort. The lower income groups had a higher estimated probability of not having an e-ID than the highest income group. Also, income support recipients were more likely to lack an e-ID. Not having a device to access the internet (smartphone, tablet or computer) was a very strong indicator of not having an e-ID; the difference was considerable, 47% points. Among those who did not speak Finnish or Swedish, the risk of not having an e-ID was 21% points higher compared to those with advanced local language skills.

**Table 2:** Average marginal effects (AMEs) based on the logistic regression model (Outcome: No e-ID). Standard errors are estimated with the delta method.

	Bivariate associations				Full model			
	AMEs	p-value	95% CI		AMEs	p-value	95% CI	
**Sex**								
Male	Ref.	.	.	.	Ref.	.	.	.
Female	0.02	0.53	-0.04	0.07	0.03	0.23	-0.02	0.08
**Age**								
50–64	Ref.	.	.	.	Ref.	.	.	.
65–93	0.21	0.00	0.14	0.27	0.08	0.02	0.02	0.15
**Monthly household income**								
Less than 1000€	0.25	0.00	0.17	0.33	0.12	0.01	0.04	0.21
1000–1499€	0.16	0.00	0.09	0.23	0.09	0.02	0.01	0.18
1500–2499€	0.07	0.04	0.00	0.14	0.05	0.22	-0.03	0.13
over 2500€	Ref.	.	.	.	Ref.	.	.	.
**Income support**								
Non-recipient	Ref.	.	.	.	Ref.	.	.	.
Recipient	0.16	0.00	0.09	0.22	0.07	0.04	0.00	0.13
Missing	0.18	0.03	0.02	0.35	0.07	0.27	-0.05	0.19
**Education in Finland**								
No	Ref.	.	.	.	Ref.	.	.	.
Yes	-0.12	0.00	-0.17	-0.06	-0.03	0.31	-0.10	0.03
**Higher education in the country of origin**								
No	Ref.	.	.	.	Ref.	.	.	.
Yes	-0.03	0.33	-0.09	0.03	0.02	0.42	-0.03	0.08
**Personal smartphone, tablet, or laptop**								
Yes	Ref.	.	.	.	Ref.	.	.	.
No	0.63	0.00	0.50	0.75	0.47	0.00	0.28	0.66
**Length of residence**	0.00	0.65	0.00	0.00	0.00	0.09	0.00	0.01
**Finnish citizenship**								
Yes	Ref.	.	.	.	Ref.	.	.	.
No	0.09	0.00	0.03	0.15	0.00	0.92	-0.06	0.07
**Local language skills (FIN/SWE)**								
Cannot speak	0.34	0.00	0.21	0.46	0.21	0.03	0.02	0.40
Basic level	0.10	0.00	0.03	0.16	0.04	0.26	-0.03	0.12
Advanced level	Ref.	.	.	.	Ref.	.	.	.
Missing	0.09	0.18	-0.04	0.23	-0.04	0.37	-0.13	0.05

## Discussion

Finland, as many other welfare states is dedicated to fast speed digitalisation of health services. While this might create easier access for many residents, not all people benefit from this equally, and some may even face exclusion from the services that should be universal in principle. Our results showed that approximately 21% of older Russian-speaking migrants reported that they did not have e-ID, which is required for the use of health and social care and many other public services. The proportion of people who did not have e-ID was much higher among the oldest age group: approximately 35% of people who were 65 or older reported not having an e-ID while among 50 to 64-year-olds the proportion was 14%. In comparison to existing evidence among the Finnish general population, 20% of Russian-speakers aged 55 to 74 years and 48% of over 74-year-olds did not have an e-ID. In the general Finnish population, these figures are 11% and 43% [37]. However, we should note that both the provision and the use of online services have been increased as a result of the COVID-19 pandemic. Our data are from the pre-pandemic time (spring 2019).

### Strengths and Limitations

The post-pandemic context creates a need for understanding the social consequences of health service digitalisation. The case of Finland provides an interesting example of the rapid digitalisation of public services in a Northern European welfare state. While our data focus on one language group only, they are based on a unique, population-based survey. The data do not include comparison group of older Finnish-born adults, but we provided comparable results by age group. Our study participants were often highly educated and Russian-speakers are considered as better integrated to the Finnish society than some other migrant groups, such as people with Somalian or Kurdish backgrounds [43]. The problems faced by our study participants suggest that the digital access problems may be even more severe in other migrant groups. For instance, older migrants from Somalia are reported to have high levels of illiteracy [44], which poses a serious challenge for the use of digital services. Regarding national comparisons, the number of ageing migrants is relatively small in Finland, which might incur less political pressure on inclusive service development, if compared to other Western European countries. Thus, there is a need for comparative research.

## Conclusions

Among older Russian-speaking migrants, older age, poorer economic situation and lack of local language skills were associated with a higher risk of not having an e-ID. This means that people who are already in a more vulnerable position also face difficulties in accessing health and social welfare services. Our results additionally showed a link between not speaking local languages and not having an e-ID, which resonates with prior knowledge on the strong status quo of the Finnish banks in providing e-IDs. To guarantee universal welfare and social rights, services should be available also in other formats than exclusively digitally. Furthermore, people who cannot access online banking details should be provided with alternative, low-cost and easy to use options for electronic identity authentication.

## Data Availability

The data presented in this study are available on request from the corresponding author. The data are not publicly available due to general data protection guidelines.
